# Successful Preventive Treatment of Oncogenic Transforming HPV Infections in Low-Grade Cytology (ASC-US/LSIL) Patients with an Adsorptive and Antioxidant Vaginal Gel

**DOI:** 10.3390/jcm12124142

**Published:** 2023-06-20

**Authors:** Attila Louis Major, Ivanna Mayboroda, Alexandra Riger

**Affiliations:** 1 Femina Gynaecology Centre, 1205 Geneva, Switzerland; 2 Department of Obstetrics and Gynecology, Urgench Branch of Tashkent Medical Academy, Urgench 220100, Uzbekistan; 3 Hospital of Yverdon-les-Bains, 1400 Yverdon-les-Bains, Switzerland

**Keywords:** atypical squamous cells of undetermined significance (ASC-US), low-grade squamous intraepithelial lesion (LSIL), cervical intraepithelial neoplasia (CIN), abnormal cervical smear findings, persistent high-risk HPV infection, p16/Ki-67 biomarker, adsorptive and antioxidative vaginal gel, silicon dioxide, sodium selenite, non-destructive medical treatment, tumor environment

## Abstract

Objective: This study aimed to investigate the preventive effect of a vaginal gel on p16/Ki-67-positive abnormal cytological cervical findings (ASC-US, LSIL) and hr-HPV in women. Methods: The study included 134 women with p16/Ki-67-positive ASC-US or LSIL. Participants were selected from a randomized controlled trial that focused on women with histological diagnoses of p16-positive CIN1 lesions or CIN2. In the treatment group (TG), 57 patients applied the vaginal gel daily for three months, while 77 patients in the “watchful wait” control group (CG) received no treatment. The study’s endpoints were cytological development, p16/Ki-67 and hr-HPV clearances. Results: At three months, cytopathological results improved in 74% (42/57) of patients in the TG, compared with 18% (14/77) in the CG. Progression occurred in 7% (4/57) of TG patients compared with 18% (14/77) of CG patients. The p16/Ki-67 status changed statistically significantly in favor of the TG (*p* < 0.001), with 83% (47/57) becoming negative, compared with 18% (14/77) in the CG. The prevalence of hr-HPV decreased significantly in the TG by 51%, and by 9% in the CG (*p* < 0.001). Conclusions: Topical application of the gel resulted in statistically significant clearance of hr-HPV and p16/Ki-67 concomitant with amelioration of cytological findings, thus providing effective prevention and protection against oncogenic development. Trial registration: ISRCTN11009040, on 10 December 2019.

## 1. Introduction

Persistent high-risk human papillomavirus (hr-HPV) infection in the genital area is causally involved in 99.7% of cervical cancer cases [1]. About 10% of hr-HPV persists for two years and is involved in oncogenic transformation. This transformation leads to the development of invasive cancer in one third of precancerous lesions such as HSIL (high grade squamous intraepithelial lesion) [2]. This underscores the importance of new tools to predict and prevent oncogenic development of persistent hr-HPV early with well-tolerated topical vaginal treatments. Without such an approach, partial surgical amputation of the cervix, such as conization, is often indicated as standard treatment, and may lead to overtreatment. The oncogenic transformation by persistent hr-HPV is a very slow process that can take up to 20 years, providing a great opportunity for medical treatment and prevention [2].

Regrettably, there is currently no recommended treatment available during the observation period after a gynecological examination indicating a low-grade lesion. This therapy-free interval, which adheres to international guidelines [3], may cause anxiety and, in some cases, high levels of psychological stress [4].

Studies indicate that chronic inflammation triggers oxidative stress and consequently alterations in viral and host genomes [5,6,7]. This allows hr-HPV to integrate into the host, a crucial step for the cervical epithelium to undergo malignant transformation [5,6,7]. Oxidative damage of DNA appears to be a multistep process in the development of CIN2 and CIN3 compared with normal epithelium [8]. Infection and inflammation-induced oxidative stress play crucial roles in the development of malignancy, yet they also present opportunities for novel treatment options [9]. For this reason, early treatment of high-risk women with an antioxidant and adsorbent topical medical device may be a good option to prevent progression in an oncogenic environment.

Given the potential for serious complications from invasive interventions, new guidelines are needed to suggest more conservative approaches during this period [10,11]. Consequently, the watch and wait timeframe provides an opportunity to use non-destructive treatments that can potentially promote lesion regression and prevent unnecessary surgeries. During this period, numerous guidelines recognize cytology and HPV detection as screening methods. However, the HPV test is not specific enough to screen for precancerous lesions. Thus, available screening tools have limited advantages.

Huber et al. emphasized that only 10–20% of women exhibit the persistent infection required for cervical carcinogenesis [12]. Therefore, it is important to select these patients. Demarco et al. showed that ASC-US associated with hr-HPV+ has an increased cumulative five-year risk for CIN2+ of 19%, which should be taken into consideration for prevention [13]. In a follow-up study over a 12-year period, including 7482 women with NILM and hr-HPV testing over an interval of two years, the risk of CIN3+ was 47.4% for persistent HPV 16 [14]. The clinical goal is to treat patients with carcinogenic risk as soon as possible as a preventive measure to achieve regression, stop progression, and avoid overtreatment. Hence, it is crucial to utilize specific biomarkers to identify cases of ASC-US/LSIL and CIN1. Simultaneous detection of the tumor suppressor protein p16 and proliferation protein Ki-67 in the same cell is considered a cytological marker for the induction of oncogenic transformation in cells of the genital tract [15]. Increased expression of HPV E7 oncoprotein induces overexpression of p16 and is associated with persistent HPV infection [16,17].

The effectiveness and safety of DeflaGyn^®^ vaginal gel were demonstrated in a randomized controlled study that utilized p16 [18]. This was confirmed by a post hoc subgroup analysis of hr-HPV- and p16/Ki-67-associated abnormal cervical cytology findings [19]. All women were analyzed for p16/Ki-67, which has excellent specificity, better than cytology or HPV [18,19,20,21,22,23].

The main objective of the present analysis is to compare various screening methods for cervical smears, including the p16/Ki-67 dual-stain test, standard cytology (Bethesda), and hr-HPV detection. A secondary analysis will assess treatment efficacy based on cytological findings, including p16/Ki-67.

The study is expected to provide valuable insights into the capacity of the well-tolerated vaginal gel DeflaGyn^®^ to prevent oncologic development in patients with ASC-US/LSIL findings who are at increased risk.

## 2. Materials and Methods

### 2.1. Study Group

Out of the total of 216 women who participated in the randomized, controlled, prospective study, and had histologically proven p16 positive CIN1 or CIN2, a group of 134 patients with ASC-US/LSIL and positive p16/Ki-67 dual-stain cytology was selected. Patients ranged in age from 25 to 60 years [18].

### 2.2. Treatment

The investigation included a three-month intravaginal treatment with DeflaGyn^®^ vaginal gel and three-month follow-up. The vaginal gel was provided by DEFLAMED International s.r.o Prague, Czech Republic. As active ingredients, the gel contained highly dispersed silicon dioxide (10.0 mg), citric acid (24.8 mg), and elemental selenium (0.25 mg) per application (5 mL). For the treatment, 5 mL of the gel was inserted deep into the vagina once daily by the patient using a vaginal applicator to dose the gel.

The ability of silicon dioxide to adsorptively bind proteins, lipoproteins, lipids, viruses, and bacteria is supported by several publications [24,25,26,27]. With an average particle size of 300 µm, the adsorbing effect of silicon dioxide is extremely high. The main component of DeflaGyn^®^ vaginal gel is DEFLAMIN^®^, which has an important antioxidant capacity. This antioxidant capacity, i.e., a willingness to transfer electrons to other atoms and molecules, is quantified in the so-called “reduction potential” (standard redox potential). Selenite has a low redox potential of +0.366 Volts in an alkaline milieu (systemically) but increases its potential to give off electrons dramatically up to −0.740 Volts in an acidic environment [28]. DeflaGyn^®^ vaginal gel has the capacity to adsorb, neutralize, and eliminate pathogenic agents such as bacteria, viruses, or potentially irritating particles from the vaginal secretions.

With a pH of 3 this vaginal gel has an acidic character, which also has the potential to inactivate HIV and other viruses and inhibit bacteriosis [29,30]. A low pH maintains the composition and concentration of bacteria in the vaginal microflora. The combined disappearance of lactobacilli and increased pH correlates with a 100–1000-fold increase in bacterial concentration of the vaginal ecosystem [31].

In contrast, control group patients did not receive active treatment and were observed using the watch and wait policy, in accordance with present recommendations.

### 2.3. Methods

Conventional cervical smears were used for cytology and p16/Ki-67 analysis. The first smear slide underwent routine cytological screening analysis, stained according to Papanicolaou and analyzed in accordance with the Bethesda system. A second slide from the same collected sample was analyzed for immunocytochemical changes using p16/Ki-67 dual biomarker technology (CINtec^®^ Plus Cytology, Roche, Switzerland).

Material from cervical smear samples additionally underwent screening for hr-HPV. For transport and cell preservation, Roche Cell Collection Medium was used. A total of 14 genotypes of hr-HPV DNA, including hr-HPV 16 and 18 and other high-risk genotypes (31, 33, 35, 39, 45, 51, 52, 56, 58, 59, 66, 68), were identified using the Roche Cobas 4800 HPV test.

### 2.4. Data Evaluation and Statistics

Remission or regression was evaluated in the cytological smear after three months of using the adsorbent/antioxidant vaginal gel or in the control group that merely underwent watchful waiting. Improvement was defined as cytological regression, an initial ASC-US, LSIL lesion that resolved or transformed to a lower status (for example, LSIL to ASC-US, ASC-US to NILM, etc.). This was evaluated after three months of treatment and a further three months of follow-up. Outcomes were ranked in decreasing order of carcinogenic risk: HSIL, ASC-H (atypical squamous cells—cannot exclude HSIL), LSIL, ASC-US [32]. Furthermore, cytological progression was recorded from low-risk (ASC-US, LSIL) to high-risk (ASC-H and HSIL). Two additional endpoints were adopted: alteration in p16/Ki-67 dual-stain cytology (CINtec^®^ Plus test) after three and six months, and disappearance of hr-HPV at three months [18].

IBM SPSS Statistics for Windows, Version 25.0. Armonk, NY, USA: IBM Corp, was used for statistical procedures. The two-tailed Fisher’s exact test was employed to compute any associated significances between groups and changes in cytological samples, HPV disappearance, and p16/Ki-67 outcomes. Evaluation of descriptive data was performed using Microsoft Office Excel (Microsoft Excel 2019, Version 1808 (Build 10399.20000), Microsoft: Wien, Austria).

## 3. Results

A total of 134 patients, 57 patients in the TG and 77 patients in the CG, with ASC-US/LSIL cytology and a positive test result for p16/Ki-67 were eligible for subgroup analysis (Figure S1 Supplementary Material).

The baseline characteristics of the study collective are presented in Table 1.

Several differences in demographic data could be observed between treatment and control groups at baseline (Table 1). In the biopsies, CIN2 was found to be significantly more common in the TG (22/57 patients) than in the CG (6/77 patients). The distribution of cytopathogical results, however, was comparable between both groups (*p* = 0.697).

At baseline, there were significantly more IHC p16-positive patients in the TG. Additionally, patients in the CG were slightly older, but this was not statistically significant (*p* = 0.152).

**Figure 1:**
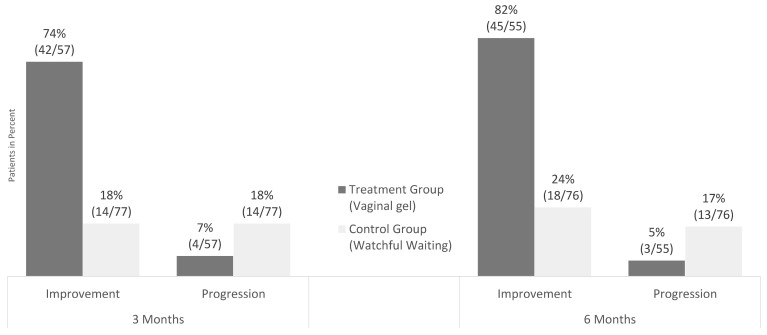
ASC-US and LSIL: IMPROVEMENT and PROGRESSION (Preventive Effect): Cytological Results after 3 and 6 months. All patients had ASC-US or LSIL and p16/Ki-67 dual-stain positivity at baseline. In total, 42 of 57 patients (74%) in the treatment group showed remission (evolution from ASC-US to NILM and from LSIL to NILM) or regression (LSIL to ASC-US) at 3 months. In the control group, 14 of 77 patients (18%) showed improvement. Progression occurred in 4/57 patients (7%) in the treatment group and in 14 of 77 (18%) in the watch and wait group at 3 months. At 6 months, 45 of 55 patients (82%) showed improvement in the treatment group and 3 of 55 patients (5%) showed progression. In the control group 18 of 76 patients (24%) improved and 13 of 76 of patients (17%) showed progression at 6 months. After 3 and 6 months, significant differences regarding Improvement, Persistence and Progression between the groups (treatment vs. control) could be shown (*p* < 0.001; Fisher´s Exact Test). ASC-US: atypical squamous cells of undetermined significance, LSIL: low grade squamous intraepithelial lesion. Improvement = Remission and Regression. Persistence not displayed.

After three months of vaginal gel application, cytological regression of 74% (42/57 patients) was significantly higher (*p* < 0.001) in the TG than in the CG with 18% (14/77 patients) (Figure 1).

A similar difference was still evident after six months. Of the patients, 82% (45/55) in the TG and 24% (18/76) in the CG experienced regression or remission (Figure 1). The difference in improvement between the groups was statistically significant when analyzed as a yes/no dichotomy (Fisher’s two-sided exact test; *p* < 0.001), and after categorization as progression, persistence, regression, and remission (Pearson’s chi-squared test; *p* < 0.001).

**Figure 2:**
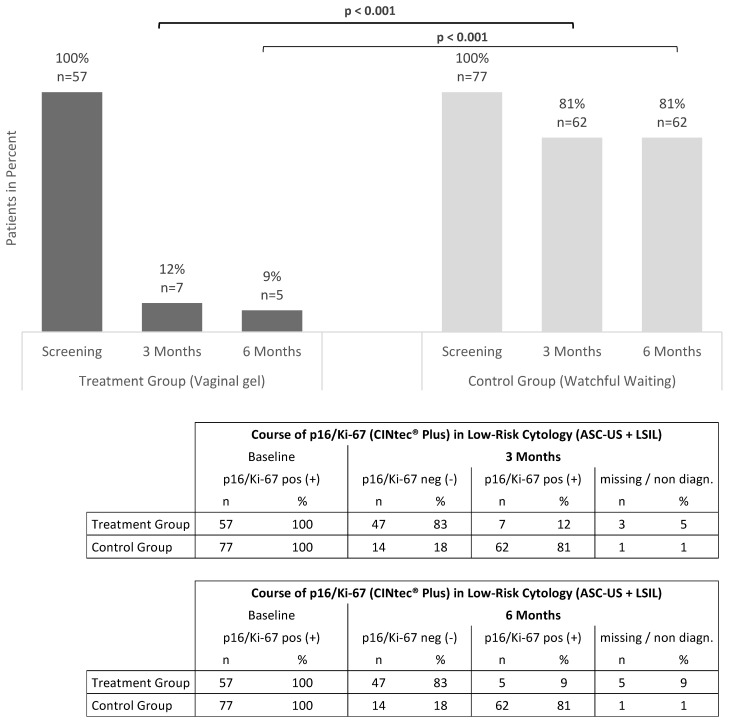
Evolution of p16/Ki-67 in ASC-US and LSIL patients. All patients were p16/Ki-67 dual-stain positive (CINtec^®^ Plus Test) at screening. The bars after 3 months and after 6 months show the proportion of patients who remained p16/Ki-67 dual-stain positive. The group comparison between treatment (vaginal gel) and control (watch and wait) is indicating a significant difference between the groups after 3 and 6 months. *p* < 0.001, Fisher’s Exact Test.

After the three-month period, 12% (7/57) of patients initially positive for the p16/Ki-67 dual-stain test in the TG remained positive, while 83% (47/57) became negative (Figure 2). Of 77 patients initially positive in the CG, 81% (62/77) remained positive, while 18% (14/77) became negative after three months (*p* < 0.001). The TG had a significantly higher proportion of women testing negative for p16/Ki-67 than the CG, as determined by Fisher’s two-sided exact test (*p* < 0.001).

Results from p16/Ki-67 dual-stain testing at six months were comparable to those at three months (Figure 2). In the TG, 83% (47/57) of p16/Ki-67 positive patients initially became negative, whereas in the CG, only 18% (14/77) of positive patients became negative. At six months, there was a statistically significant difference in p16/Ki-67 dual-stain findings between the two groups (Fisher’s two-sided exact test; *p* < 0.001).

When combined with ASC-US and LSIL data, the vaginal gel had a highly significant impact on individuals who were initially p16/Ki-67 dual-stain positive. Only 12% of patients (7/57) in the TG were still p16/Ki-67 dual-stain positive after three months, compared with 81% (62/77) the CG. After three months, individuals with low-grade cytological changes in the TG had a p16/Ki-67 clearance rate of 83% (47/57) compared with 18% (14/77) the CG. Data from three patients in the TG and from one patient in the CG were missing (Figure 2).

**Figure 3:**
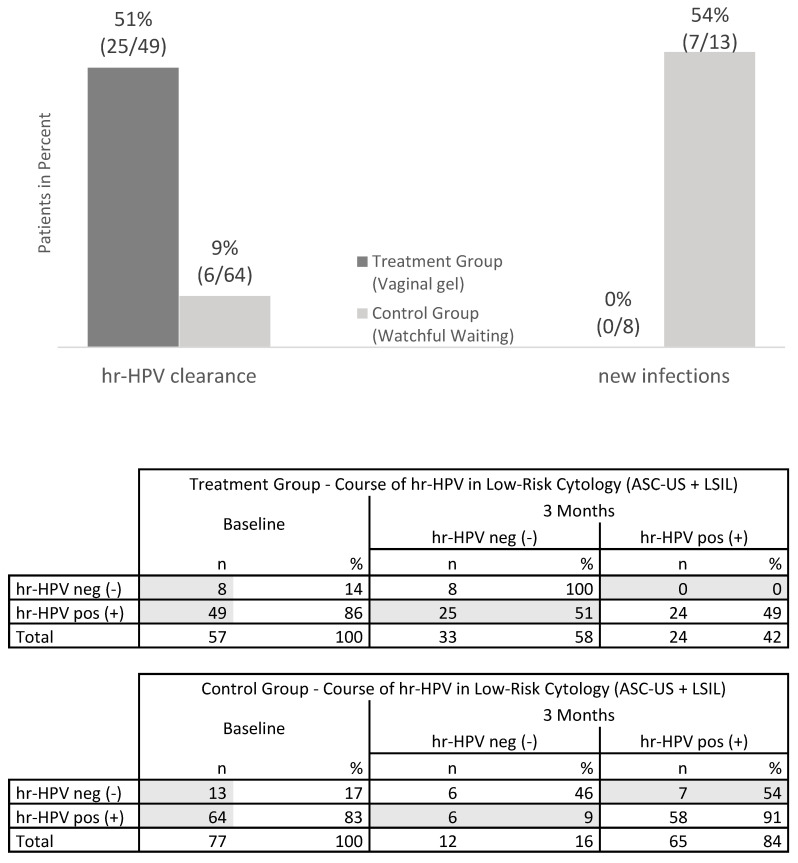
hr-HPV clearance and new hr-HPV infections after 3 months of treatment or watchful waiting. At screening, 49 patients in the treatment group, all ASC-US or LSIL, were hr-HPV positive. At 3 months, 25/49 patients (51%) in the treatment group showed hr-HPV clearance. In the control group, only 6 of 64 patients (9%) showed hr-HPV clearance. At baseline 8 patients in the treatment group were hr-HPV negative. None of these patients progressed to hr-HPV positivity. In 7 of 13 patients (54%), who were hr-HPV negative at baseline in the control group, new hr-HPV infection occurred after 3 months. All patients in both groups were p16/Ki-67 dual-stain positive at baseline. Clearance of hr-HPV is significantly higher in the treatment group vs. in the control group (*p* < 0.001; Fisher’s Exact Test). n = number, Clearance = change from hr-HPV positive to negative, new infections = change from hr-HPV negative to positive; ASC-US: atypical squamous cells of undetermined significance, LSIL: low grade squamous intraepithelial lesion, hr-HPV: high risk HPV.

In the TG, 86% (49/57) of patients had positive hr-HPV tests at baseline (Figure 3). Of them, 58% (33/57) tested negative for hr-HPV at three months, resulting in a hr-HPV clearance of 51% (25/49) in the TG. In contrast, of 83% (64/77) of patients in the CG who had positive hr-HPV tests at baseline, 84% (65/77) were positive after three months. Only 9% (6/64) of those who were initially positive in the CG cleared the infection, whereas 54% (7/13) of those who were initially negative developed a new infection. Notably, there were no new hr-HPV infections in the TG among patients who were initially negative at screening (0/8).

These results indicate a statistically significant reduction in hr-HPV prevalence in the TG compared to the CG after three months (*p* < 0.001, Fisher’s two-sided exact test).

No serious adverse events were observed and none of the minor adverse events required discontinuation of the device application [18].

## 4. Discussion

### 4.1. Summary of the Main Results

The effectiveness of a preventive non-invasive topical application to the cervix and vagina in women with ASC-US/LSIL who tested positive for p16/Ki-67 and hr-HPV is demonstrated in this study. This was apparent despite the fact that the number of CIN2 women was significantly higher in the vaginal gel group at the beginning of the trial (Table 1). This study is the first to utilize p16/Ki-67 and hr-HPV as biomarkers for evaluating the efficacy of topical treatments to prevent oncogenic development of ASC-US/LSIL.

This study found that the topical treatment was effective in preventing carcinogenic transformation in women ASC-US/LSIL positive for p16/Ki-67 and for hr-HPV. Administration of the gel led to a statistically significant improvement in treatment success, with a 74% increase in regression of cytopathological findings in the TG compared with 18% in the CG. Additionally, the gel significantly reduced disease progression by 7% in the TG compared with 18% in the CG. These findings were consistent with cytopathological observations and were previously demonstrated in a histological and colposcopic study [18]. Since significantly more CIN2 occurred in the TG (39% vs. 8%), the treatment success further emphasizes its effect. The significant reduction of hr-HPV (from 49 to 24 women in the TG) and p16/Ki-67 (from 57 to 7 positive tests) in comparison with the CG also supported these findings. Moreover, three months after the end of vaginal gel application, the improvement was still evident, with an 82% regression rate in the TG versus 24% in the CG and a 5% progression rate in the TG compared with 17% in the CG, as illustrated in Figure 1.

### 4.2. Results in the Context of Published Literature

Previously, a retrospective data analysis demonstrated the safety and tolerability of the adsorptive and antioxidant vaginal gel. Furthermore, the analysis indicated a positive impact on abnormal cytological findings [33]. The efficacy and safety of the vaginal gel to treat women with colposcopy and histopathologically proven cervical lesions were observed recently by Major et al. [18]. These findings were supported by a post hoc subgroup analysis of hr-HPV- and p16/Ki-67-associated abnormal cervical cytology findings, including HSIL [19].

The now published study shows that the vaginal gel is effective in treating patients with ASC-US/LSIL tested positive for p16/Ki-67. Administration of the gel resulted in significant treatment success with increased cytological regression and elimination of hr-HPV.

A crucial aspect of the approach adopted is the high specificity of p16/Ki-67 for abnormal cytological findings and CIN. It is worth noting that ASC-US/LSIL has a 75.2% specificity for CIN3 in p16/Ki-67-positive women. In smears that are negative for both hr-HPV and p16/Ki-67, the risk of CIN3 was 1.2%, compared with 15.6% if HPV-positive. When both hr-HPV and p16/Ki-67 tests are positive, the risk of CIN3 rises to 27% [34].

Another study found that the specificity of both p16/Ki-67 alone and cytology for detecting CIN2+/VAIN2+ was 95%, whereas the specificity of HPV alone was only 41.6%. Moreover, p16/Ki-67 was the best test for detecting suspicious cervico-vaginal disease and preventing under-evaluation [20]. In a study involving patients with ASC-US, p16/Ki-67 immunocytochemistry showed the highest specificity for detecting high-grade dysplasia, with rates of 74.2% in CIN3+ and 82.5% in CIN2+. These rates were significantly better than those observed for HPV [21]. Moreover, p16/Ki-67 dual-stained cytology testing reduced the number of referrals for colposcopy and was more sensitive and specific than HPV in detecting most cases of CIN3 in LSIL patients [22]. Selection of p16/Ki-67 and hr-HPV-positive women performed better than cytology and decreases needless colposcopies and biopsies [23,35,36].

This is probably the first published data demonstrating the effective treatment of high-risk p16/Ki-67 ASC-US and LSIL patients and prevention of oncogenic development with a topical gel during the watchful waiting period. Numerous studies have been conducted using medical treatments for precancerous lesions, but they have not been conclusively successful, either because of significant side effects or low efficacy.

It is worth mentioning two randomized, controlled trials that utilized applications of imiquimod with self-administered vaginal suppositories [37,38]. The imiquimod group demonstrated complete histologic remission in 37% of patients (19 out of 51), in contrast to 84% of patients who underwent LLETZ (large loop excision of the transformation zone) [38]. Another trial that studied the topical administration of imiquimod for CIN2+ had to be canceled due to the difficulty in finding patients to take part in the trial [39]. Imiquimod has been shown to be effective for topical treatment of CIN2 and CIN3, but is inappropriate for preventive treatment of oncogenic development due to local and systemic side effects [40].

Studies with the vaginal gel suggest that it has therapeutic potential as it impacts oncogenic progress, as measured by p16/Ki-67. The results demonstrate that the gel can prevent and even annihilate the carcinogenic effect of HPVs, at least for a while, by influencing the vaginal microenvironment and probably as a consequence, the modulation of the local microbiome and immunity [41,42].

The main goal to avoid overtreatment is to find the right balance between surgical and preventive conservative treatment of the cervix. By selecting ASC-US/LSIL patients with specific biomarkers, efficient prevention of a vaginal gel associated with no or only few side effects can be attained during the watchful waiting phase. By not using this window, physicians risk losing the opportunity to decrease the amount of partial cervical amputations with its associated complications and side effects. Preventive treatment during the watchful waiting time may not only decrease or hinder invasive development but may sustain, and by that shorten, the duration of spontaneous regression.

Studies with larger sample sizes and of a longer duration will be required to verify the potential of the vaginal gel on the prevention of precancerous and cancerous lesions of the cervix.

### 4.3. Strengths and Weaknesses

The strengths of this study are that results were derived from patients with histopathology of CIN2 and p16-positive CIN1 and that p16/Ki-67 dual-stain cytology was used to select ASC-US and LSIL. p16/Ki-67 is a marker of oncogenic transformation and is therefore an indirect marker of persistent hr-HPV infection. The weaknesses of the study include the small sample size and the fact that the data were a subgroup analysis rather than a randomized clinical trial. Furthermore, longer follow-up studies are required to accurately further evaluate the effect of the adsorptive and antioxidant vaginal gel on preventing the development of cervical cancer.

### 4.4. Implications for Practice and Future Research

Long term follow-up data over a period of ten years or more show that in patients with persistent hr-HPV, the risk of CIN3+ increases impressively, even in NILM and ASC-US/LSIL. By early selection of high-risk patients with the oncomarker p16/Ki-67 or in the future with a specific epigenetic methylation marker, a topical treatment to restore an immunologically strong vaginal milieu is a good preventative strategy to inhibit oncogenic transformation. This is especially important for young women who do not want to risk any impairment of their fertility or experience preterm delivery. Treatment with an effective vaginal gel with a low side effect profile should be suggested in situations where there is a higher risk of oncogenic transformation, such as persistent hr-HPV infections or p16/Ki-67-positive tests. Even postponing the need for surgical intervention would be a benefit for all women, but especially for fertile women.

Investigations with the vaginal gel are continuing in further trials to build on the evidence already presented.

## 5. Conclusions

ASC-US and LSIL selected by persistent hr-HPV or by an oncogenic marker, such as p16/Ki-67, indicate increased risk of developing precancerous lesions or cancer at a later date. Modifying the vaginal environment with a topical treatment to eliminate hr-HPV and early reversal of oncogenic transformation may prevent development of cervical cancer. A vaginal gel with adsorptive and antioxidant properties applied locally for three months impressively removed oncogenic HPVs and blocked their effect on the oncomarker p16/Ki-67. The preventive treatment was associated with improved cytological results and effectively protected against oncogenic development, at least temporarily. Therefore, by providing an efficient non-surgical treatment with a low side effect profile, the gel (DeflaGyn^®^ vaginal gel) could be suitably indicated during the watch and wait period.

Overlooking this window of opportunity may result in a missed chance to reduce the number of partial cervical amputations with their associated complications and consequences. As a conservative medical treatment during the watch and wait period, the gel may also reduce invasive development by increasing the early regression rate.

## Figures and Tables

**Table 1:** Baseline characteristics of p16/Ki-67 dual-stain positive patients with ASC-US or LSIL cytology (*n* = 134).

		Treatment Group (*n* = 57)		Control Group (*n* = 77)		*p*
Age (years) Mean ± SD		32.98	±6.78	35.16	±8.60	0.152 ^+^
Relevant gynecological history **		2	3.51%	16	20.78%	0.004 ^x^
Smoking		18	31.58%	17	22.08%	0.237 ^x^
HPV vaccination		10	17.54%	12	15.58%	0.816 ^x^
Histology ^§^	CIN1	35	61.40%	71	92.21%	<0.001 ^x^
	CIN2	22	38.60%	6	7.79%	
	Total	57	100.00%	77	100.00%	
Cytology	ASC-US	17	22.70%	20	20.60%	0.697 *
	LSIL	40	53.30%	57	58.80%	
	Total	57	100.00%	77	100.00%	
High-risk HPV ^§§^	Yes	49	85.96%	64	83.12%	0.811 ^x^
	No	8	14.04%	13	16.88%	
	Total	57	100.00%	77	100.00%	
p16/Ki-67 ^§§§^ (CINtec^®^ Plus)	CIN1	35	61.40%	71	92.21%	<0.001 ^x^
	CIN2	22	38.60%	6	7.79%	
	Total	57	100.00%	77	100.00%	
IHC p16 ^§§§§^	CIN1	30/35	85.71%	37/71	52.11%	<0.001 *
	CIN2	22/22	100.00%	6/6	100.00%	
	Total	52/57	91.23%	43/77	55.84%	
High-Risk HPV	CIN1	29/35	82.86%	58/71	81.69%	n.a.
	CIN2	20/22	90.91%	6/6	100.00%	
	Total	49/57	85.96%	64/77	83.12%	

Values given as mean, ± standard deviation, %; Statistical analysis: + Wilcoxon rank-sum test; ^x^ Fisher’s two-sided exact test; * Pearson chi-squared test; ** Relevant gynecological history (conservative surgeries of the uterus and surgeries for adnexal diseases); ^§^ CIN1 p16 positive (IHC or CINtec^®^ Plus); ^§§^ HPV Cobas 4800 test; ^§§§^ CINtec^®^ Plus Roche (p16/Ki-67); ^§§§§^ CINtec^®^ Roche (p16) Histology-Test; n.a. not analyzed; ASC-US: atypical squamous cells of undetermined significance; LSIL: low-grade squamous intraepithelial lesion.

## Data Availability

All datasets presented in this study are included in the article. Further inquiries can be directed to the corresponding author.

## Supplementary Materials

The following supporting information can be downloaded at: https://www.mdpi.com/article/10.3390/jcm12124142/s1, Figure S1: Consort flow diagram of patients included in the study.
