# Supplementary material for: Successful Preventive Treatment of Oncogenic Transforming HPV Infections in Low-Grade Cytology (ASC-US/LSIL) Patients with an Adsorptive and Antioxidant Vaginal Gel
Source: J Clin Med. 2023 Jun 20;12(12):4142. doi: 10.3390/jcm12124142 (PMC10299567; doi:10.3390/jcm12124142)
Supplement: Supplementary file 1 [file jcm-12-04142-s001.zip › jcm-2432094-supplementary.pdf]

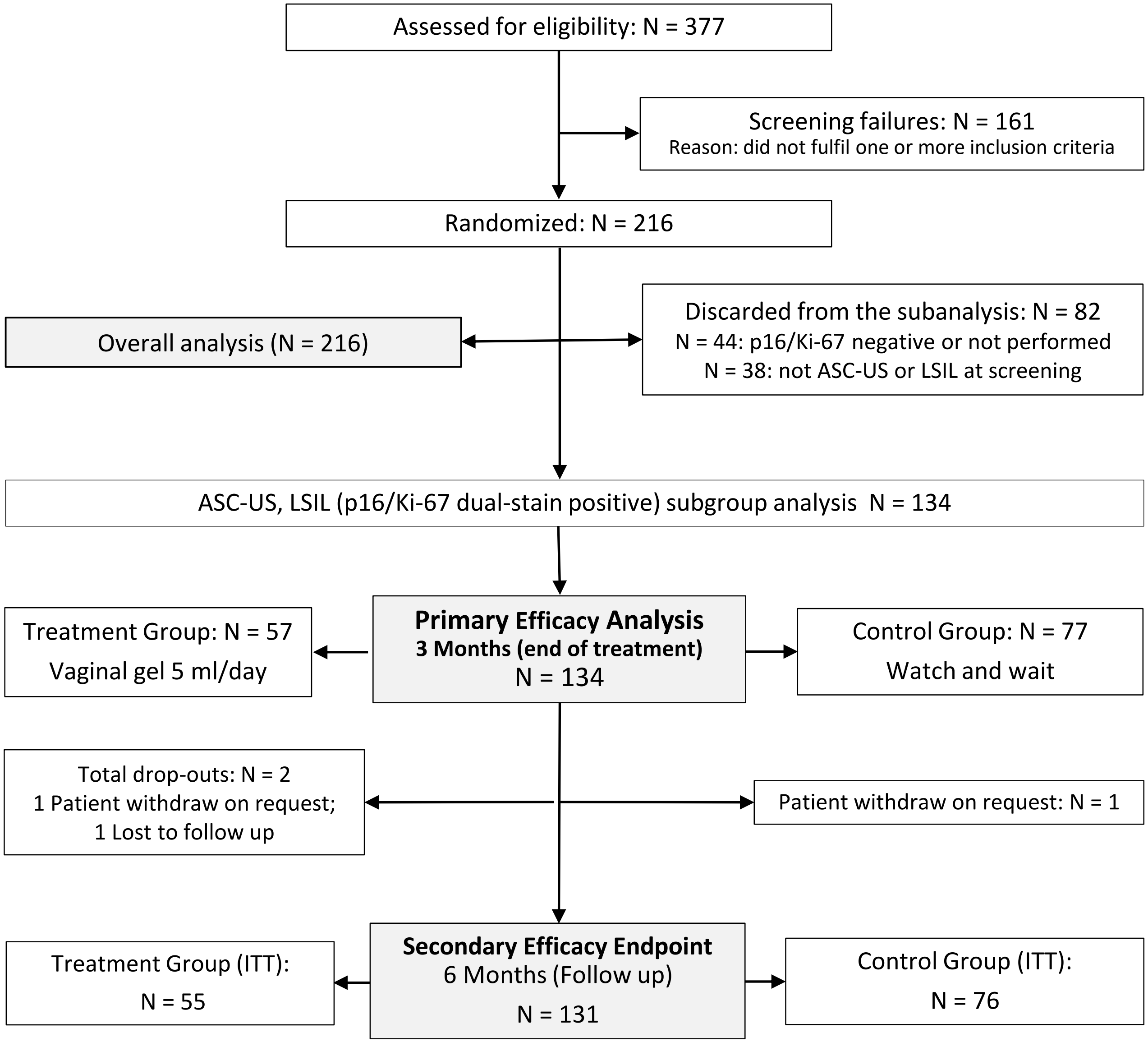

Figure S1: CONSORT Flow Diagram of patients included in the study ITT: Intention-To-Treat Population
